# Visual search and real-image similarity: An empirical assessment through the lens of deep learning

**DOI:** 10.3758/s13423-024-02583-4

**Published:** 2024-09-26

**Authors:** Marco A. Petilli, Francesca M. Rodio, Fritz Günther, Marco Marelli

**Affiliations:** 1 Department of Psychology, University of Milano-Bicocca, Milano, Italy; 2 Institute for Advanced Studies, IUSS, Pavia, Italy; 3 Department of Psychology, Humboldt University at Berlin, Berlin, Germany; 4 NeuroMI, Milan Center for Neuroscience, Milan, Italy; 5 Department of Brain and Behavioral Sciences, University of Pavia, Pavia, Italy

**Keywords:** Visual search, Visual similarity, Perceptual processing, Convolutional neural networks, Search efficiency, Computer vision

## Abstract

The ability to predict how efficiently a person finds an object in the environment is a crucial goal of attention research. Central to this issue are the similarity principles initially proposed by Duncan and Humphreys, which outline how the similarity between target and distractor objects (TD) and between distractor objects themselves (DD) affect search efficiency. However, the search principles lack direct quantitative support from an ecological perspective, being a summary approximation of a wide range of lab-based results poorly generalisable to real-world scenarios. This study exploits deep convolutional neural networks to predict human search efficiency from computational estimates of similarity between objects populating, potentially, any visual scene. Our results provide ecological evidence supporting the similarity principles: search performance continuously varies across tasks and conditions and improves with decreasing TD similarity and increasing DD similarity. Furthermore, our results reveal a crucial dissociation: TD and DD similarities mainly operate at two distinct layers of the network: DD similarity at the intermediate layers of coarse object features and TD similarity at the final layers of complex features used for classification. This suggests that these different similarities exert their major effects at two distinct perceptual levels and demonstrates our methodology’s potential to offer insights into the depth of visual processing on which the search relies. By combining computational techniques with visual search principles, this approach aligns with modern trends in other research areas and fulfils longstanding demands for more ecologically valid research in the field of visual search.

## Introduction

**Fig. 1:**
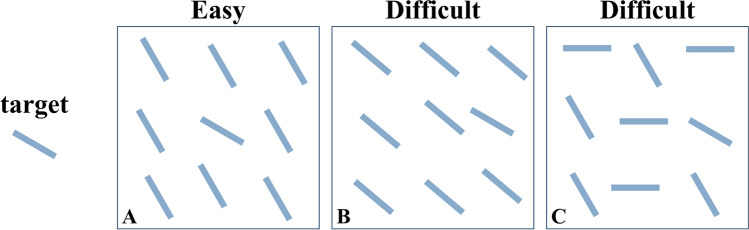
Impact of target–distractor and distractor–distractor similarity on search efficiency. *Note.* Finding a left-tilted line becomes more difficult as TD similarity increases (A vs B) or DD similarity decreases (A vs C)

Visual search literature shows that our ability to find a target stimulus heavily depends on the similarity relationships between the elements in the scene (see Fig. 1). Based on a wide range of findings, Duncan and Humphreys (1989) demonstrated that search performance varies continuously across tasks and conditions and identified two key factors that impact it: the similarity between targets and distractors (TD), where an increase results in a decrease in performance (e.g., Foster & Westland, 1995; Nagy & Sanchez, 1990; Verghese & Nakayama, 1994), and the similarity among distractors (DD), where an increase leads to an improvement in efficiency (e.g., Farmer & Taylor, 1980; Feldmann-Wüstefeld et al., 2017; Rosenholtz, 2001). These two factors would interact in affecting performance, scaling each other’s effects.

Although both TD and DD are grounded on relationships of similarity, they rely on distinct processes. Duncan and Humphreys (1989) suggested that DD effects arise through widespread suppression of distractors grouped by similarity, whereas TD was hypothesised to operate through a matching process between the input and the target template. In a more recent perspective, TD effects have been linked to the increasing difficulty of the visual periphery in simultaneously comparing all items to the target when TD similarity is high (Buetti et al., 2016, 2019). Conversely, DD effects have been linked to interitem interactions produced by high DD similarity, which facilitate distractor rejection (Lleras et al., 2019; Z. J. Xu et al., 2021).

Significant research has been conducted since Duncan and Humphreys (1989), leading to a more nuanced understanding of the impact of similarity patterns. This includes the identification, at various similarity levels, of different types of functions that relate response times (RTs) to set size, each targeting distinct underlying search mechanisms (see Haslam et al., 2001; Lleras et al., 2022). Nevertheless, the principles introduced by Duncan and Humphreys still offer a robust framework for understanding the impact of similarity relationships in visual search. However, there are some critical aspects to consider.

First, while TD and DD similarity effects are confirmed in a wide range of published results, they remain ‘a general, somewhat approximate summary of a wide range of search findings’ (Duncan & Humphreys, 1989, p. 444) with no systematic attempt to quantitative measure stimulus similarity.

Second, empirical findings supporting the validity of the similarity principles are mostly limited to a small domain of stimulus materials using very basic stimuli and extremely limiting constraints (such as letters or colour patches varying in a limited number of attributes). While these paradigms offer precise control over perceptual variables, they fail to capture the richness of real-world scenarios. In everyday life, looking for an object entails directing attention towards an entity of multidimensional attributes among others that might unpredictably share some of them. Given these considerations, the gap between lab-based studies and real-world scenarios is so significant that it is problematic to generalise lab-based findings to everyday visual searches (Wolfe, 2020).

Third, using basic stimuli inevitably limits our understanding of the level of representation at which similarity operates. In other words, when we search for a lost pencil box, do we rely primarily on a low-level representation encoding its basic attributes (e.g., colour, size) or the complex object representation based on its whole design? Investigating similarity effects by means of basic stimuli cannot contribute to answering this question.

The first aim of this study is to offer an updated investigation of the impact of similarity in visual search using real images as stimuli. Other studies have already tested similarity effects using real images (e.g., Alexander & Zelinsky, 2011, 2012; Trapp & Wienrich, 2018). Still, their approach did not allow a comprehensive account of these effects because they (1) did not simultaneously and orthogonally manipulate DD and TD similarity, (2) the similarity was not measured on a continuous gradient, and (3) visual search was investigated using limited sets of images (e.g., images of app icons or teddy bears). However, ‘alterations in search efficiency can only be understood by considering both variables together’ (Duncan & Humphreys, 1989, p. 442), and a continuous estimate of similarity is required to accurately represent changes in the search performance on a continuous gradient.

With these aims, we adopted a pioneering approach that leverages the capabilities of deep convolutional neural networks (DCNN) to quantitatively estimate the similarity between images (Günther et al., 2023). Once trained, DCNNs take an image as input and produce the object’s label presented in the image as output. At their core, DCNNs convert images to high-dimensional feature maps. The similarity between these maps provides a similarity estimate between the corresponding images. Notably, the validity and psychological plausibility of similarity estimates obtained in this way have been empirically demonstrated in various experiments in the cognitive domain (Günther et al., 2023; Petilli et al., 2021; Zhang et al., 2018). In the context of this study, adopting such an approach makes it possible to measure TD and DD image similarity and evaluate their effect on search performance.

Besides that, we also explored which level of feature representation underlies the effects of TD and DD similarity. Previous literature suggests that various levels of visual representation contribute to visual search. At the visual processing level, Lleras et al. (2022) describe visual search as a dynamic process that handles both coarse and detailed object representations provided by parallel peripheral and serial foveal processing. At the memory level, Wolfe (2020) theorises the existence of both low- and high-level internal representations of the target: one is the ‘guiding template’, a coarse representation incorporating task-relevant features guiding attention towards likely targets (Kerzel, 2019; van Loon et al., 2017). The second is the ‘target template’, a precise representation (Bravo & Farid, 2014) to be matched to attended items to ascertain, for example, that a given pencil box is actually mine and not just similar to mine.

Given this, one might wonder: If visual search relies on different levels of representation, on which of them do TD and DD similarity operate? Are they acting upon different stages of visual processing? To address these questions, similarity principles become crucial if we test them at different levels of stimulus representations to reveal which best explains search behaviour.

**Fig. 2:**
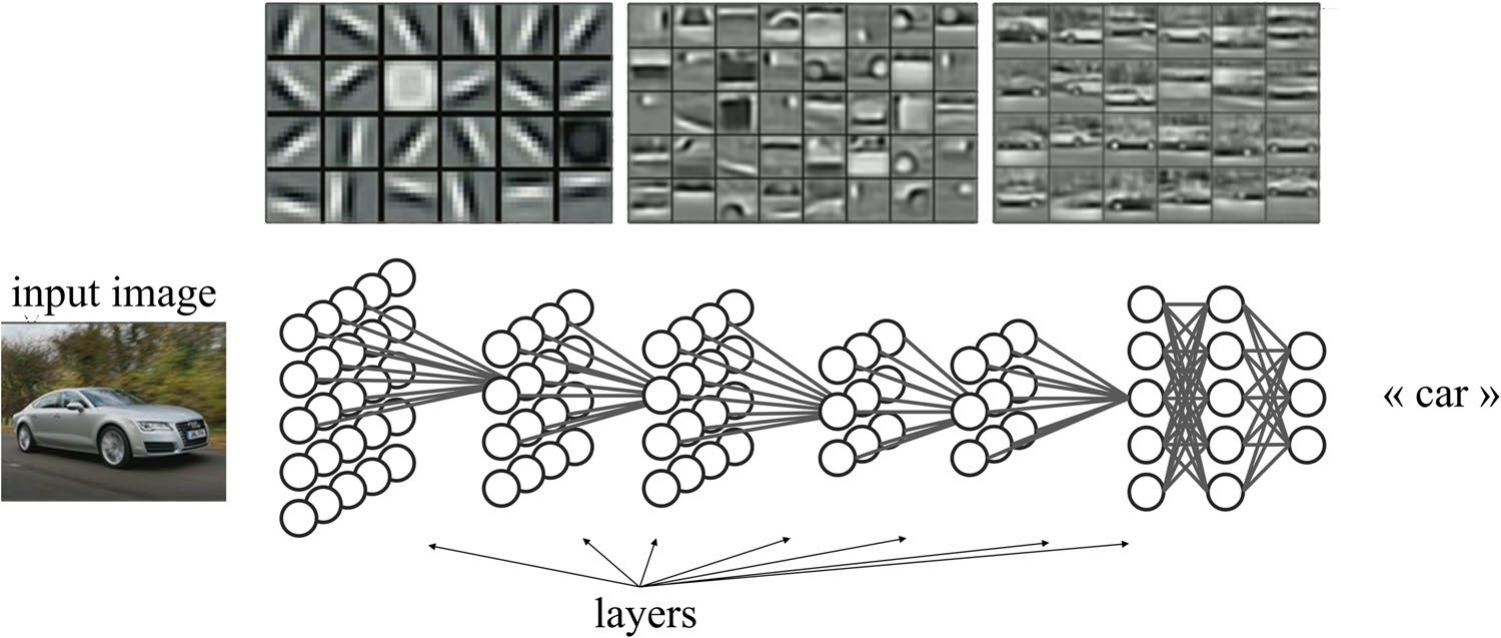
Hierarchical organization of internal layers in a deep convolutional neural network. *Note.* Simplified representation of the internal architecture of a DCNN along with a visualisation—the top—of learned features in progressively deeper layers (sourced from Lee et al., 2011)

Importantly, DCNNs offer a valid framework to formalise such aspects, providing a numerical characterization of image features at hierarchically organised layers with increasing complexity (see Fig. 2). These internal representations, although differing in many respects from human ones, still offer one of the best approximations of the progress of visual information processing along the human ventral stream (for some reviews, see Celeghin et al., 2023; Lindsay, 2021). As we move deeper and approach the output of DCNN, the low-level features are progressively integrated into more complex patterns, reaching the final DCNN layers that represent the image through high-level features, such as the complex representation of the entire object to be classified (Kriegeskorte, 2015; Zeiler & Fergus, 2014). To identify the level of representation at which similarity affects search, we analysed TD and DD effects at the various DCNN layers and tested which best explains search behaviour. In the context of this study, this issue can be rephrased as the following question: Is the similarity between basic or complex features that matters in TD vis-à-vis DD similarity effects?

## Methods

### Computational framework

The present study exploits DCNNs for image classification to extract vector representations for individual images (Krizhevsky et al., 2012). DCNNs are brain-inspired computational models designed for object recognition that are very popular in computer vision (LeCun et al., 2015). Despite some parallels, DCNNs process visual information in ways that diverge from human visual processing in many respects. For example, compared with humans, DCNNs are less robust to image corruption (Geirhos, Temme, et al., 2018), do not seem to learn object detectors at the level of single units (Gale et al., 2020), are not as good at integrating global object shapes (Baker et al., 2018; Jarvers & Neumann, 2023), and are more biased towards textures and colours rather than using shape as a primary cue for object recognition (Geirhos, Rubisch, et al., 2018; Landau et al., 1988). Overall, DCCNs are far from faithfully reproducing human visual processing. Nevertheless, DCNNs still learn internal representations that roughly approximate human and nonhuman primate inferotemporal cortex representations (Khaligh-Razavi & Kriegeskorte, 2014; Kriegeskorte, 2015). The validity of these representations is more evident when considering their capacity to approximate relative differences (i.e., similarities) among alternative external objects (i.e., second-order isomorphism; Roads & Love, 2023) as shown, for example, in studies using representational similarity analyses revealing that activation patterns in DCNNs match those in brain areas involved in object identification (e.g., Kalfas et al., 2018; Kriegeskorte, 2015; Yamins et al., 2014). This was confirmed at the behavioural level, showing that the DCNN similarity estimates not only align with human ratings of visual similarity (Günther et al., 2023; Jozwik et al., 2017; Petilli et al., 2021; Zhang et al., 2018) but also outperform such ratings in predicting human behaviour in perceptual tasks (Günther et al., 2023).

The internal architecture of DCNNs consists of a multilayer structure composed of numerous dimensions. Each of these dimensions is activated by certain visual features. The dimensions can be seen as axes of a multidimensional visual space of features, and each value can be considered a coordinate. Thus, the vector encoding these dimensions can be interpreted as a point within such a visual space, and the proximity between points as the visual similarity between the corresponding images (with images visually similar—i.e., with a similar combination of visual features—ending up being mapped to nearby points in the space).

**Fig. 3:**
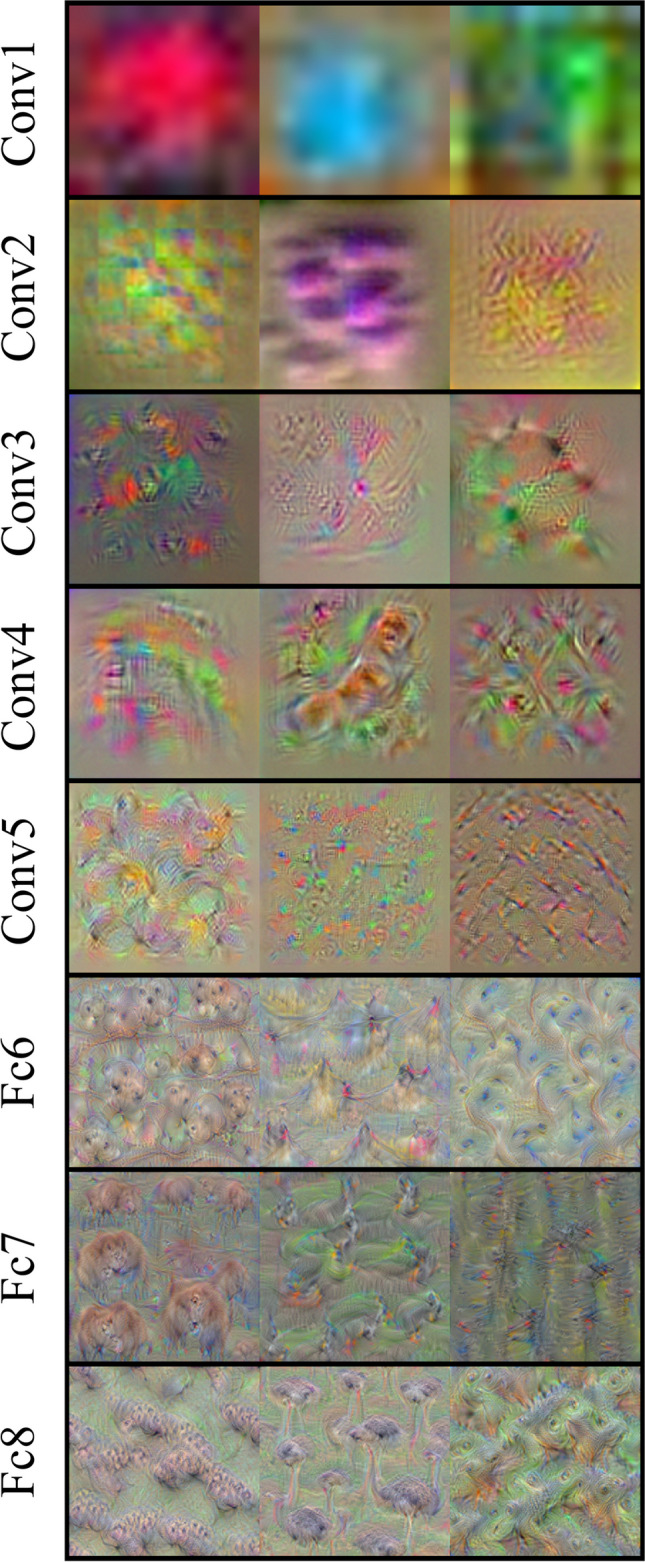
Feature visualisation in progressively deeper layers of a deep convolutional neural network. *Note.* Visualisation of distinct features learned by each layer of a DCNN. From graphical inspection, initial layers detect low-level features that are integrated into more complex features proceeding through the network. Feature visualisation has been conducted using the deepDreamImage function in MATLAB. (Colour figure online)

In this study, we used as DCNN the pretrained network VGG-F (Chatfield et al., 2014) available in the MatConvNet MATLAB library (Vedaldi & Lenc, 2015). The VGG-F model is a streamlined variant of the VGG models (from the Visual Geometry Group at the University of Oxford), including seven layers (five convolutional—conv1, conv2, conv3, conv4, conv5—and two fully connected—fc6, fc7—before the classification layer fc8^1^). The activations in these layers represent the set of features in the image, with initial layers naturally encoding low-level features (e.g., edges, colour patterns) and final layers naturally encoding high-level patterns combining basic features into texture and complex object shapes similar to humans (Khaligh-Razavi & Kriegeskorte, 2014; Kriegeskorte, 2015; Zeiler & Fergus, 2014; but see Celeghin et al., 2023; Y. Xu & Vaziri-Pashkam, 2021; see Figs. 2 and 3).

Here, as an estimate of vision-based similarity between two images, we calculated the cosine of the angle between their vectors within a DCNN layer (Günther et al., 2023). Target and distractor stimuli were selected based on similarity relationships between activation vectors induced by the ‘fc6’ layer consisting of 4,096 dimensions, which has been shown to outperform the other layers in predicting reaction times in various tasks, including perceptual, such as image discrimination and visual priming (Günther et al., 2023). Similarity estimates derived from this layer were also initially employed to test the effect of TD and DD similarity on search performance. In a second set of analyses, we considered similarity based on all seven layers for both TD and DD similarity and tested which layers better captured search performance.

### Participants

Forty participants were involved in the study (33 females, *M*_age_ = 22.6 years, *SD* ± 3.2) via the Sona-System, an online platform used for participant enrolment (https://milano-bicocca.sona-systems.com), in exchange for course credits. If available, participants were allowed to participate in multiple experimental sessions on different days, with the condition that each participant would encounter a different set of blocks and stimuli in every session, thus ensuring that no stimuli were presented more than once to any participant. In total, the participants contributed to 51 experimental sessions. The study only included participants who indicated they had a normal or corrected-to-normal vision. The study was approved by the minimal-risk committee of the Department of Psychology of the University of Milano-Bicocca (Prot. N, RM-2023–656) and was run according to the principles of the Declaration of Helsinki.

In the present study, statistical analyses were performed at the item level with generalised additive models (GAMs) and continuous predictors, diverging from conventional search experiments, and thus lacking specific sample size references. The initial sample size determination was established following Brysbaert and Stevens (2018) recommendations for item-level analyses using linear mixed-effects models, and additional data were collected to minimise the risk of statistical power issues as we moved from linear models to GAMs. In linear models, continuous predictors and two-level factors require estimating the same number of parameters. Considering the effect of interest in our study included two continuous predictors in interaction (DD Similarity × TD Similarity), this is equivalent to a 2 × 2 factorial design. Following Brysbaert and Stevens’s recommendation of at least 1,600 observations per condition, a total of 6,400 observations would be recommended. Here, we collected data for 51 experimental sessions, each contributing with 384 observations, resulting in 19,584 observations (for target-absent [TA] and target-present [TP] trials each), thus largely exceeding Brysbaert and Stevens’s (2018) recommendations to minimise the risk of low statistical power.

### Material

Stimuli were selected from ImageNet (Deng et al., 2009), a large-scale database containing images labelled for categories. Only square images were considered and resized at a standard resolution of presentation (i.e., 224 × 224 pixels, which corresponds to the resolution of the image input for the VGG-F model). Images with frames or readable text were excluded.

**Fig. 4:**
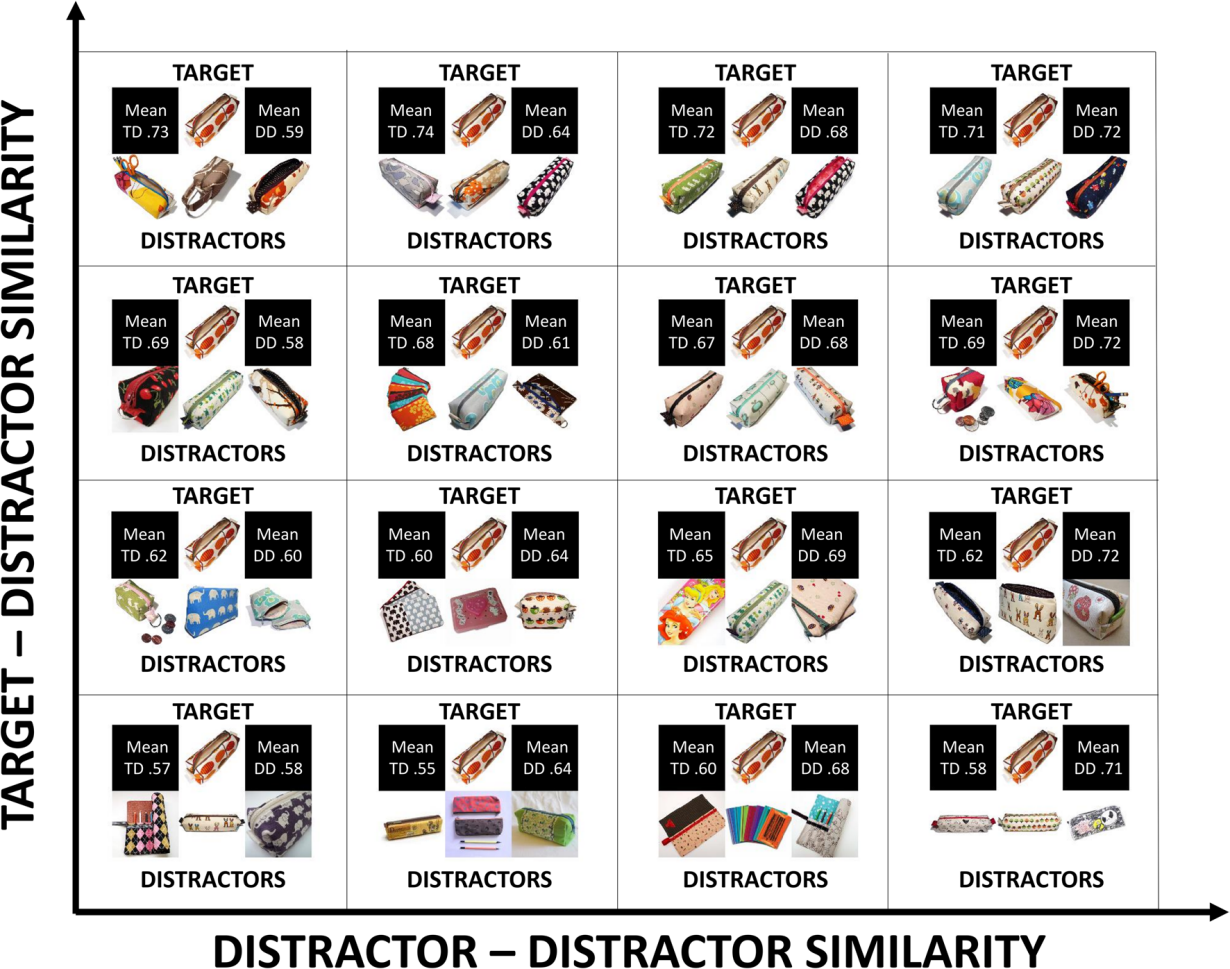
Example of stimuli from the ImageNet category ‘Pencil box’. *Note.* Each quadruplet of images in the boxes corresponds to a specific combination of target (the top image) and distractors (the three images under the target). The number on the top-left black quadrant corresponds to the mean similarity between the target and the distractors as estimated through DCNN. The number on the top right black quadrant corresponds to the mean similarity between distractors as estimated through DCNN. (Colour figure online)

The experiment included 24 visual search blocks, each consisting of 49 image stimuli taken from a distinct category of ImageNet, randomly chosen. This approach of selecting from heterogeneous categories was aimed at maximising the variability in relevant features across the stimuli. Figure 4 shows an example of stimuli taken from the ImageNet category ‘Pencil box’. As can be seen, this category tends to predominantly emphasise the long-axis orientation of the objects as a key feature for differentiating and recognising them. However, it is important to note that the complete range of stimuli we used in the experiment involves a much broader spectrum of relevant features. For instance, the ‘Earphone’ category tends to emphasise the curvature and shape of the objects as relevant features, while others like ‘Damask’ give more relevance to the colour and texture of the objects (for the full list of stimuli, see https://osf.io/2vad8/).

In the experiment, each block contained 96 visual search trials (50% TP and 50% TA) at different set sizes (~ 33% set size of 3, ~ 33% set size of 6, ~ 33% set size of 9). Each block included 49 image stimuli taken from a single category of ImageNet randomly chosen (i.e., one target and 48 distractors for 24 categories, resulting in 1,176 different images used in the experiment). Each box in Fig. 4 includes a set of stimuli (i.e., a specific combination of target [the top image] and distractors [the three images under the target]) that we used in the same search trial.

The target image remained the same in each block while the distractors varied to produce various TD and DD similarity levels. For each level of similarity, a set of three distinct images was selected as distractors. To this aim, we first selected a random image to be used as a target. Then, we considered all the possible combinations of triplets of images available as candidate distractors for a single search array. For each triplet, we computed the similarity between the target image and each of the three distractors (i.e., mean(cos($\overrightarrow{T},\overrightarrow{D}$_1_), cos($\overrightarrow{T},\overrightarrow{D}$_2_), cos($\overrightarrow{T},\overrightarrow{D}$_3_)) and excluded those triplets for which the difference between the maximum similarity and the minimum similarity exceeded 0.025 (max(TD similarity) − min(TD similarity) > 0.025). This criterion was applied to keep similarity between the target and each of the three distractors of a triplet relatively constant. Similarly, we computed the cosine similarity between each pair of distractors of all triplets (i.e., mean(cos($\overrightarrow{D}$_1_ $,\overrightarrow{D}$_2_), cos($\overrightarrow{D}$_1_ $,\overrightarrow{D}$_3_), cos($\overrightarrow{D}$_2_ $,\overrightarrow{D}$_3_)). Again, we excluded those triplets for which the difference between the maximum similarity and the minimum similarity exceeded 0.025 (max(TD similarity) − min(TD similarity) > 0.025). This criterion was applied to keep the similarity among distractors forming a triplet relatively constant.

**Fig. 5:**
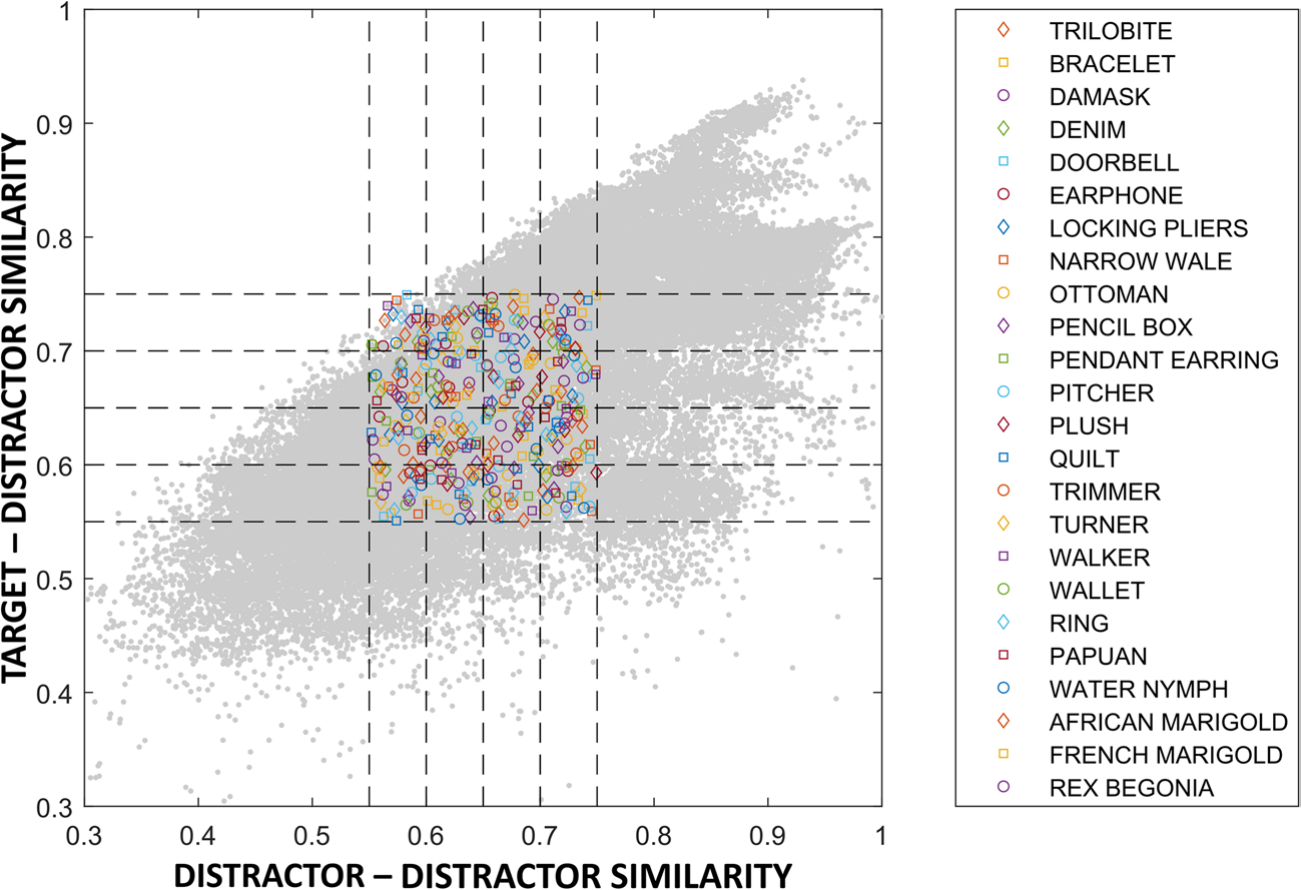
Distribution of target–distractor and distractor–distractor similarities across selected ImageNet categories. *Note.* The distribution of all combinations of TD similarity and DD similarity for the selected categories is represented in grey. Coloured marks show the distribution of the specific target–distractor combinations selected for the experiment. The legend reports the label associated with the selected category from ImageNet. (Colour figure online)

Then, the range of cosine similarity between stimuli of the experiment was set at an intermediate level, ranging from 0.55 to 0.75. These precise values were chosen based on the visual examination of the distribution of TD and DD similarity for the available target–distractor combinations. The range of similarities (both in terms of TD and DD similarity) between 0.55 and 0.75 was well covered by target–distractor combinations, as can be seen in Fig. 5. Any target–distractor combinations outside this range were excluded. Other regions were excluded because they were underrepresented and not consistently covered by all the categories. Note that such a distribution of similarities is not specific to the set of images we selected but reflects the natural occurrences of similarity relationships among objects in the real world. Indeed, some areas in Fig. 5 represent ‘impossible’ or ‘improbable’ regions to find adequate combinations of target and distractor images. The upper-left corner in Fig. 5 corresponds to an impossible area with combinations of images for which the distractors are simultaneously very similar to the target but different from each other—thus, a logically not feasible scenario. Other regions of the TD–DD distribution, instead, are severely sparsely populated. These regions correspond to the higher levels of TD or DD similarity (where the cosine is close to 1). Let us consider a multidimensional space in which the points in space are uniformly distributed, and the proximity between points denotes their similarity. The likelihood of having three equidistant points (distractors) from a central point (target) increases as the distance from the central point increases.^2^ The exclusions of these areas of similarity, therefore, not only ensure a balanced representation of the data in terms of TD and DD similarity but also align with the logical constraints of similarity relationships among objects in the real world.

To select target–distractor combinations well distributed within this area, we segmented the range of both TD and DD similarity into four bins, resulting in 16 cells. We then selected one triplet for each cell, covering the combination of four distinct TD and four distinct DD similarity levels. Thus, these levels ranged from 0.55 to 0.75 with interval increments of 0.05. As a result, each target–distractor triplets within a visual search block fell in one of the following TD and DD similarity ranges: (i) 0.55–0.60 (representing the lowest level of similarity); (ii) 0.60–0.65; (iii) 0.65–0.70; (iv) 0.70–0.75 (representing the highest level of cosine similarity).

In case a level of similarity included more than one suitable target–distractor combination, one of them was selected at random. An exception was made for the condition with the highest TD similarity (i.e., from 0.70 to 0.75) and lowest DD similarity (i.e., from 0.55 to 0.60) since this condition is underrepresented at the top-left corner (see Fig. 5). In this case, when multiple sets were available, we selected the one with the TD similarity and DD similarity closest to 0.75 and 0.55 (the top-left corner in Fig. 5) to balance the similarity distribution in the portion of the space less represented.

If there was no suitable distractor triplet for each of the 16 similarity levels, a new target was chosen, and the process was repeated iteratively. Categories for which no image combinations met this criterion were excluded. This procedure was repeated until we obtained 24 categories.

Each of the 16 target–distractor triplets was used as stimuli for the various visual search trials. Every target–distractor triplet was displayed six times in an experimental block, covering three set sizes and both target-absent (TA) and target-present trials (TP) trials, as follows: (a) a search array with three stimuli (TP trials: one target and two different distractors), (TA trials: three distinct distractors); (b) a search array with six stimuli (TP trials: one target and five distractors; i.e., one unique and two repeated twice), (TA trials: six distractors; i.e., each repeated twice); (c) a search array with nine stimuli (TP trials: one target and eight distractors; i.e., one repeated twice and two repeated three times), (TA trials: nine distractors; i.e., each repeated three times). Note that each target–distractor triplet replicates the structure of standard search experiments, where the change of reaction times (RTs) as a function of set size is typically estimated through various search trials. These trials employ the same stimuli to keep constant similarity but vary in the number of stimuli to evaluate the impact of set size on search time (Duncan & Humphreys, 1989; Wolfe, 2020).

In each trial, the search display consisted of evenly spaced images arranged on the perimeter of an imaginary circle centred on the screen and with a radius equal to 40% of the screen’s height. Starting at 12 o’clock, 12 possible stimulus positions were evenly spaced around the circle. A (randomly selected) arc of adjacent positions was used for each display, equating the distance between adjacent images across display sizes (see Duncan & Humphreys, 1989). When repeated distractor images were presented (i.e., set size of 6 and 9), they never appeared in adjacent positions to prevent strong perceptual grouping by proximity (besides similarity) of physically identical stimuli in adjacent locations as set size increased (Wagemans et al., 2012).

### Procedure

Participants were tested online using PsychoPy (Version 2021.2.3; Peirce et al., 2019) through Pavlovia (https://pavlovia.org/).

Each experiment consisted of eight blocks out of the possible 24 (each block was administered to 17 different participants).

A practice block including 16 trials (not part of the experiment) preceded the experimental blocks. The order of the experimental blocks was randomised. Additionally, within each block, the order of the trials was randomised with the constraint that the same set of distractors was never presented in consecutive trials. This approach avoided potential intertrial effects induced by distractor repetition and expectation (Petilli et al., 2020).

Before the beginning of each block, participants were shown the target image of the block. To familiarise participants with the incoming stimuli, each block began with 16 practice trials in which all target–distractor combinations belonging to the block were presented once. Then, the 96 experimental trials of the block were presented sequentially.

**Fig. 6:**
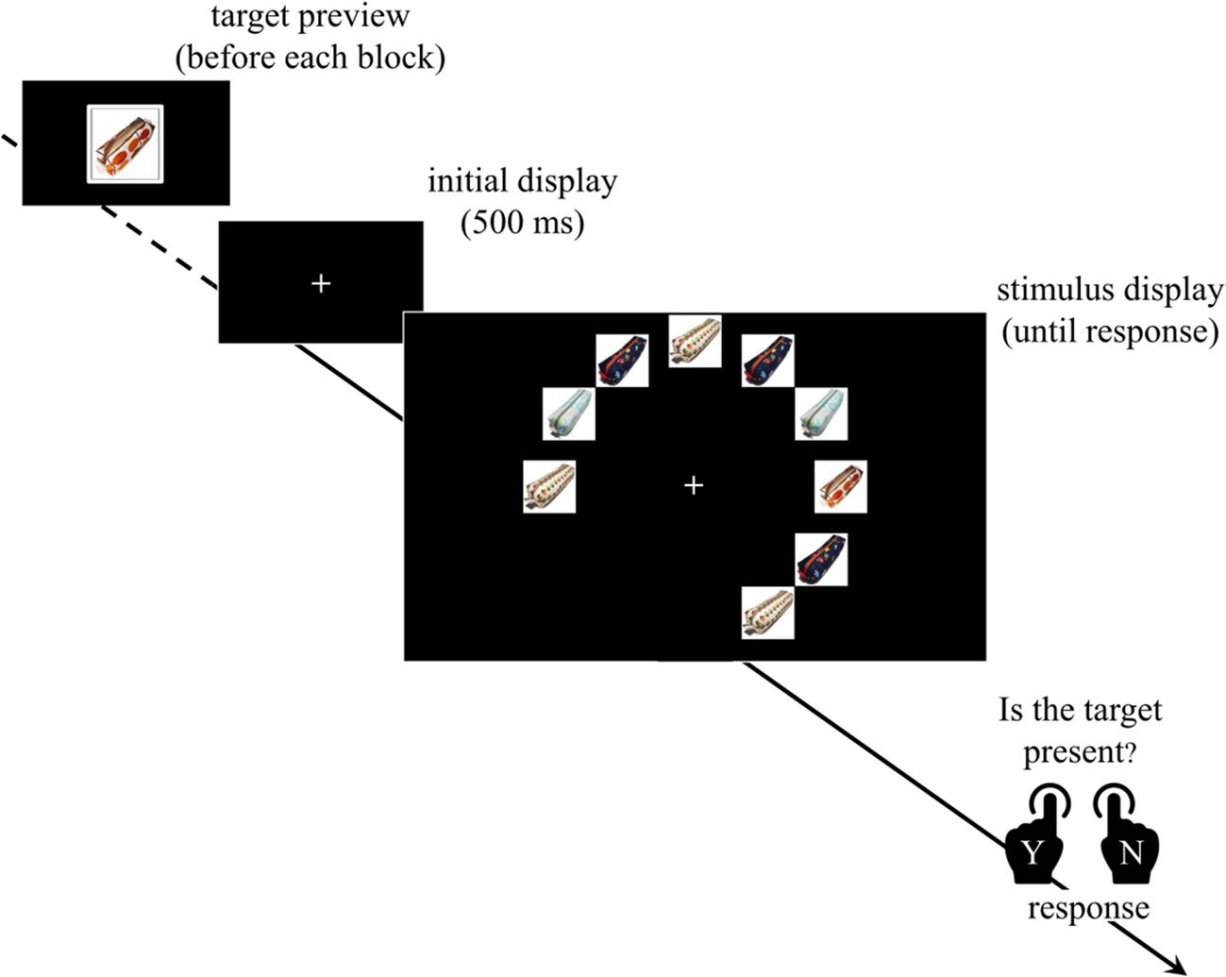
Schematic representation of the experimental timeline for the experiment. *Note*. Participants are presented with a preview of the target image before each block. Each trial begins with a 500-ms display with a central fixation cross. The stimulus display then appears, showing a circular arrangement of images. Participants respond by indicating whether the target is present or not. (Colour figure online)

Each trial began with the presentation of a central fixation cross. After a 500 ms, the search array was displayed and remained visible until a response was made. The intertrial interval had a duration of 2,000 ms. The trial procedure is illustrated in Fig. 6.

Participants were instructed to maintain central fixation before the search array appeared, and they were informed that they were free to move their eyes once the search array was displayed. Their task was to search for the target stimulus, ignore the distractors, and respond as quickly as possible, indicating whether the target stimulus was present (by pressing the ‘z’ key on the keyboard) or absent (by pressing the ‘m’ key on the keyboard). If the response was incorrect, the search display remained visible, and the feedback ‘ERROR!’ appeared along with the target in the centre of the screen. No feedback appeared for correct answers.

### Statistical analysis

To account for potential nonlinear effects, analyses were performed via GAMs using the mgcv R package (Wood, 2017; https://CRAN.R-project.org/package=mgcv). GAMs also allowed us to model search performance on a trial-by-trial basis and include random effects, accounting for variability between subjects and blocks. Preliminary tests using linear models were implemented via linear mixed-effect regression using the R packages lme4 (Bates et al., 2015) and lmerTest (Kuznetsova et al., 2017). The AIC comparisons between lmer and GAM models consistently favoured the latter (see the Supplementary Materials), indicating the suitability of using the nonlinear approach.

Trial RT was the dependent measure throughout all analyses. Trials with errors (3.17%), anticipatory responses (RTs < 100 ms; *N* = 2 trials), and delayed responses (exceeding three times the IQR above the third quartile of RT for each trial type: 2.1%) were excluded. In addition, to exclude the impact of overly influential outliers and ensure the robustness of the GAMs, data points were removed after model fitting based on a threshold of three standardised residual model errors, and the final GAMs were then refitted on the respective truncated data sets (Baayen et al., 2008).^3^ To avoid overfitting the effects of the specific stimuli used, the flexibility of the smooth terms in each GAM was constrained to k = 3. The same analyses were conducted for both TP and TA trials separately.

In the first set of analyses, we initially assessed the effect of similarity on search performance (i.e., trial RT) using TD and DD similarity estimates computed using the vector representation induced by the sixth layer (‘fc6’) of the DCNN, which has been demonstrated to generally outperform other layers in capturing human behaviour across various cognitive tasks (although the sixth and the seventh typically produces similar results and can be used quite interchangeably; Günther et al., 2023).

In a preliminary step, the model was specified as follows: As primary terms of interest, tensor product interactions were used to model all two-way interactions and the three-way interaction between our independent variables of interest—DD similarity and TD similarity—as well as the covariate set size. The model also incorporated tensor product smooths for TD similarity, DD similarity, and set size independently. As control variables (CV), the model included a smooth term for trial order (i.e., the ordinal position of each trial within its block of stimuli) to control for learning effects across trials, and a tensor product smooth interaction between the X and Y positions of the search array (i.e., computed as the average *X* and *Y* coordinates of the pair of middle items of the search array—or the middle item in odd set sizes) capturing spatial biases in stimulus positioning.^4^ The model further accounted for random effects (RE) by specifying smooth terms for participant and block as random intercepts, as well as random slopes for trial order and for the interaction of the *X* and *Y* position within participant and block, ensuring that order and spatial biases were accounted for across individuals and blocks.

For both TP and TA analyses, the three-way interaction (*p* values > 0.595), and the random slopes for the interaction of the *X* and *Y* position within participant and block (all *p* values > 0.521) were found to be not significant, so they were excluded from the final GAM to keep the model simple.

Thus, our final model can be summarised as follow:

$$\text{RT }\sim \text{ TD }:\text{ DD }+\text{ SetSize }:\text{ TD }+\text{ SetSize }:\text{ DD }+\text{ TD }+\text{ DD }+\text{ SetSize }+\text{ CV }+\text{ RE}$$

In subsequent analyses, we compared the contribution of TD and DD similarity across all the model layers to identify the best-performing in predicting trial search time. Model comparisons were performed by estimating the Akaike information criteria (i.e., AIC; Wagenmakers & Farrell, 2004), which returns an estimation of the quality of the model in terms of fit to the data. When comparing the AIC of two models, a difference in AIC (i.e., ΔAIC) equal to 2 is generally considered indicative of evidence in favour of the one with the lower AIC (Hilbe, 2011). This suggests that the model with the lower AIC is 2.7 times more likely to be better in terms of Kullback–Leibler distance from the ‘real’ distribution than the model with the higher AIC (Wagenmakers & Farrell, 2004).

Specifically, we considered the ΔAIC produced by each similarity estimate across the seven layers (i.e., TD similarity in conv1 vs conv2 vs conv3,...). This was done (separately for TP and TA trials), estimating ΔAIC produced by each TD similarity layer when added to seven distinct baseline GAMs, each including a predictor of DD similarity from one layer. The structure of the GAM baseline and full models remained consistent with previous analyses, except that the predictor of TD similarity (and its interactions) was exclusively included in the full model.

For example, to test the ΔAIC produced by TD similarity estimated in the conv1 layer of the model, we first fitted seven baseline models including DD similarity from a specific layer (i) and its interaction with set size:

$$\text{Baseline}\_\text{i}=\text{ RT }\sim \text{ SetSize }:{\text{DD}}_{\text{layer}\_\text{i}}+ {\text{DD}}_{\text{layer}\_\text{i}}+\text{ SetSize+ CV +RE}$$

Then we added to each baseline model, TD similarity from the conv1 layer and its interactions:

$$\text{Full}\_\text{i }=\text{ RT }\sim{\text{TD}}_{\text{conv}1}:{\text{DD}}_{\text{layer }\_\text{i}}+\text{ SetSize }:{\text{TD}}_{\text{conv}1}+\text{ SetSize }:{\text{DD}}_{\text{layer }\_\text{i}}+{\text{TD}}_{\text{conv}1}+{\text{DD}}_{\text{layer }\_\text{i}}+\text{ SetSize }+\text{ CV }+\text{ RE}$$

The ΔAIC produced by TD similarity from conv1 was quantified as the difference in the AIC between each full and corresponding baseline model, with more negative values indicating a higher improvement in the model quality:

$$\triangle\text{AICi}=\text{AIC}(\text{Full}\_\text{i})-\text{AIC}(\text{Baseline}\_\text{i})$$

These analyses were repeated using TD similarity estimated in each model layer, resulting in 49 ΔAIC values, with each of the seven layers of TD similarity tested against seven possible baselines of DD similarity. Finally, the ΔAIC values were compared across TD similarity layers with a linear mixed-effects model. This model included ΔAIC values as the dependent variable, the layer used for TD similarity as a predictor (i.e., from conv1 to fc7) and the layer used for DD similarity (i.e., from conv1 to fc7) as a random intercept:

$$\triangle\mathrm{AIC}\sim\mathrm{TDlayer}+\left(1\left|\mathrm{DDlayer}\right.\right)$$

Then, the same analyses were performed to compare ΔAIC as explained by DD (rather than TD) similarity from the seven layers (this time combined with baseline models including the layers of TD similarity as predictors).

## Results

**Fig. 7:**
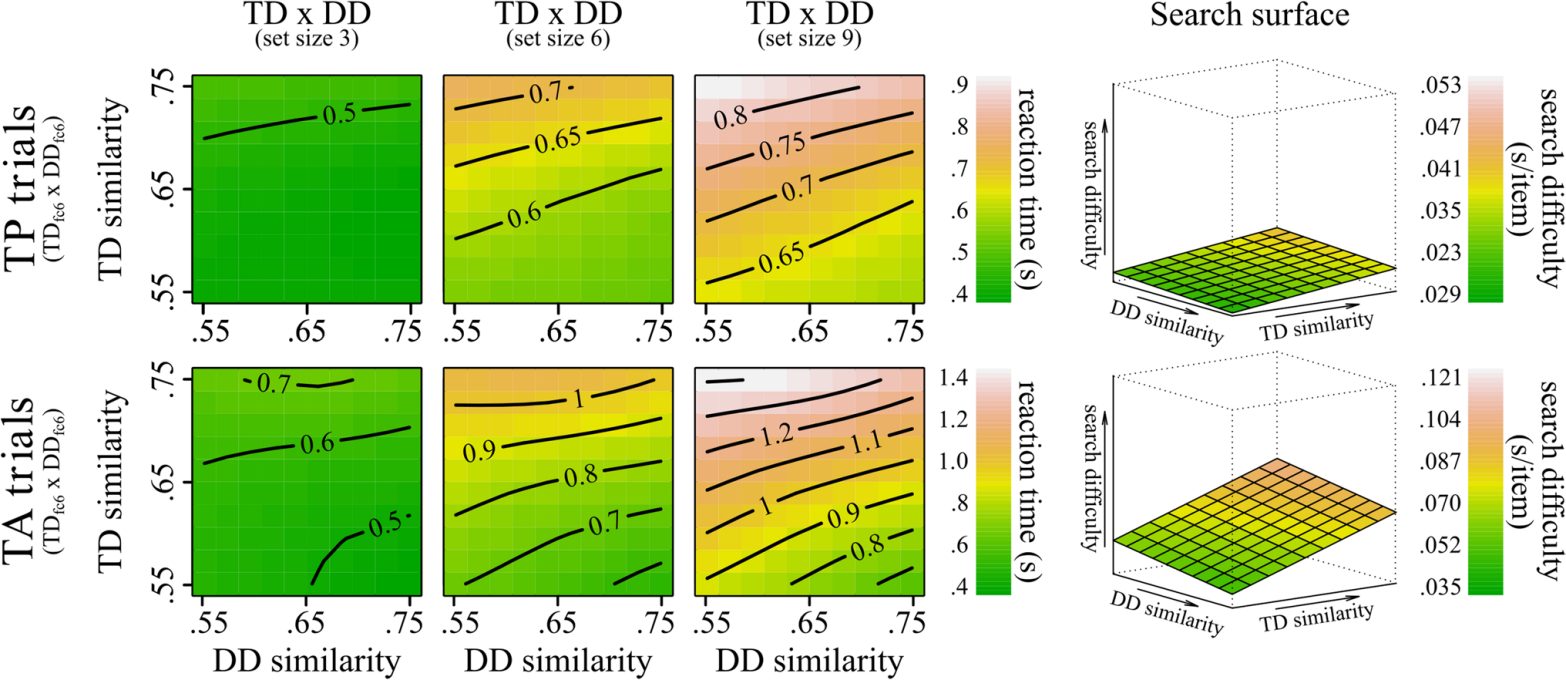
Effect of TD similarity and DD similarity on RTs as estimated by the GAMs full models. *Note*. The figure shows the effect of TD similarity and DD similarity on RTs as estimated by the GAMs full models (lines 1–2 of Table 1). The left contour plots show the effect of TD similarity and DD similarity (both calculated via DCNN’s fc6 layer) on RTs in TP (top graphs) and TA trials (bottom graphs) separately for the three set sizes. The colour gradient, from green to red, represents the increase in RTs. Each contour line denotes a specific RT value. The proximity of the lines to one another denotes the rate of change in RTs: Closer lines suggest steeper changes. To allow comparability between the effects in TA and TP trials the scales are kept consistent within the two conditions. The right 3D plots show the reconstructions of the search surface, indexing changes in search difficulty as a function of TD and DD similarity. For comparability with the seminal work by Duncan and Humphreys (1989), search difficulty was calculated as the linear slope of the fitted RT × set size function. Note, however, that while this method provides a straightforward graphical approximation of the changes in search difficulty, the linear slope is an ambiguous measure if interpreted in terms of attentional involvement (Haslam et al., 2001; Kristjánsson, 2015) as different types of search slopes (i.e., linear and logarithmic) have been linked to distinct types of visual processing (e.g., Lleras et al., 2022). Plots were generated using the vis.gam function. (Colour figure online)

First, we tested the effects of similarity on RT. Table 1 reports detailed results. The interaction between TD similarity and DD similarity was significant in TA trials. We refrained from interpreting this result, as it did not hold in TP trials and in additional analyses conducted for each set size. Besides this, the overall pattern of results in TA trials was comparable with TP trials except for substantially larger strength of the effects in the TP trials. The interactions between Set Size:TD similarity and Set Size:DD similarity were significant. Here, the rate of change in RTs increased with the increase in set size. Additional analyses separately for each set size showed the same pattern of results in all conditions. The effects of TD similarity and DD similarity were significant. As can be seen in Fig. 7, RTs consistently slowed as TD similarity increased and DD decreased.

**Table 1:** Results for the primary terms of interest from each GAM model tested

**Model**			**TD: Set Size**		**DD: Set Size**		**TD: DD**		**Set Size**		**TD**		**DD**		
		**R**^**2**^	**F**	**ΔR**^**2**^	**F**	**ΔR**^**2**^	**F**	**ΔR**^**2**^	**F**	**ΔR**^**2**^	**F**	**ΔR**^**2**^	**F**	**ΔR**^**2**^	**ƩΔR**^**2**^
TP	_**Full**_	.379	41.1^***^	.003	7.6^**^	< .001	1.1^n.s^	< .001	1046.8^***^	.012	204.3^***^	.014	37.9^***^	.001	.030
TA	_**Full**_	.527	195.1^***^	.009	21.5^***^	.001	2.9^*^	< .001	1398.9^***^	.025	1122.1^***^	.058	124.1^***^	.005	.098
TP	_**Set Size 3**_	.351	–	–	–	–	1.1^n.s^	< .001	–	–	20.6^***^	.005	6^*^	< .001	.005
TP	_**Set Size 6**_	.343	–	–	–	–	1.2^n.s^	< .001	–	–	86.3^***^	.019	8.6^**^	.001	.020
TP	_**Set Size 9**_	.337	–	–	–	–	.3^n.s^	< .001	–	–	102.3^***^	.024	16.6^***^	.002	.025
TA	_**Set Size 3**_	.417	–	–	–	–	.1^n.s^	< .001	–	–	271.8^***^	.052	60^***^	.006	.058
TA	_**Set Size 6**_	.474	–	–	–	–	2.2^n.s^	< .001	–	–	474.4^***^	.080	48.1^***^	.004	.085
TA	_**Set Size 9**_	.506	–	–	–	–	3.5^n.s^	< .001	–	–	536^***^	.085	82.4^***^	.008	.093

The table reports the results for the primary terms of interest from each GAM model tested (with TD and DD similarity calculated via DCNN’s fc6 layer). Rows 1 and 2 report results for the full models run separately for TP and TA trials. Rows 3 to 8 present results for the models run at each of the three set-size levels separately for TP and TA trials. For each model, the R^2^ value is reported to represent the model’s overall explained variance. Additionally, for each model term, the table provides the *F*-value and the change in unique variance (ΔR^2^), which indicates the improvement in explained variance provided by the inclusion of each specific term to the model. Finally, the column ƩΔR^2^ reports the sum of the unique variance of all terms of interest

*p < .05; **p < .01; ***p < .001; ^n.s.^ = nonsignificant

**Fig. 8:**
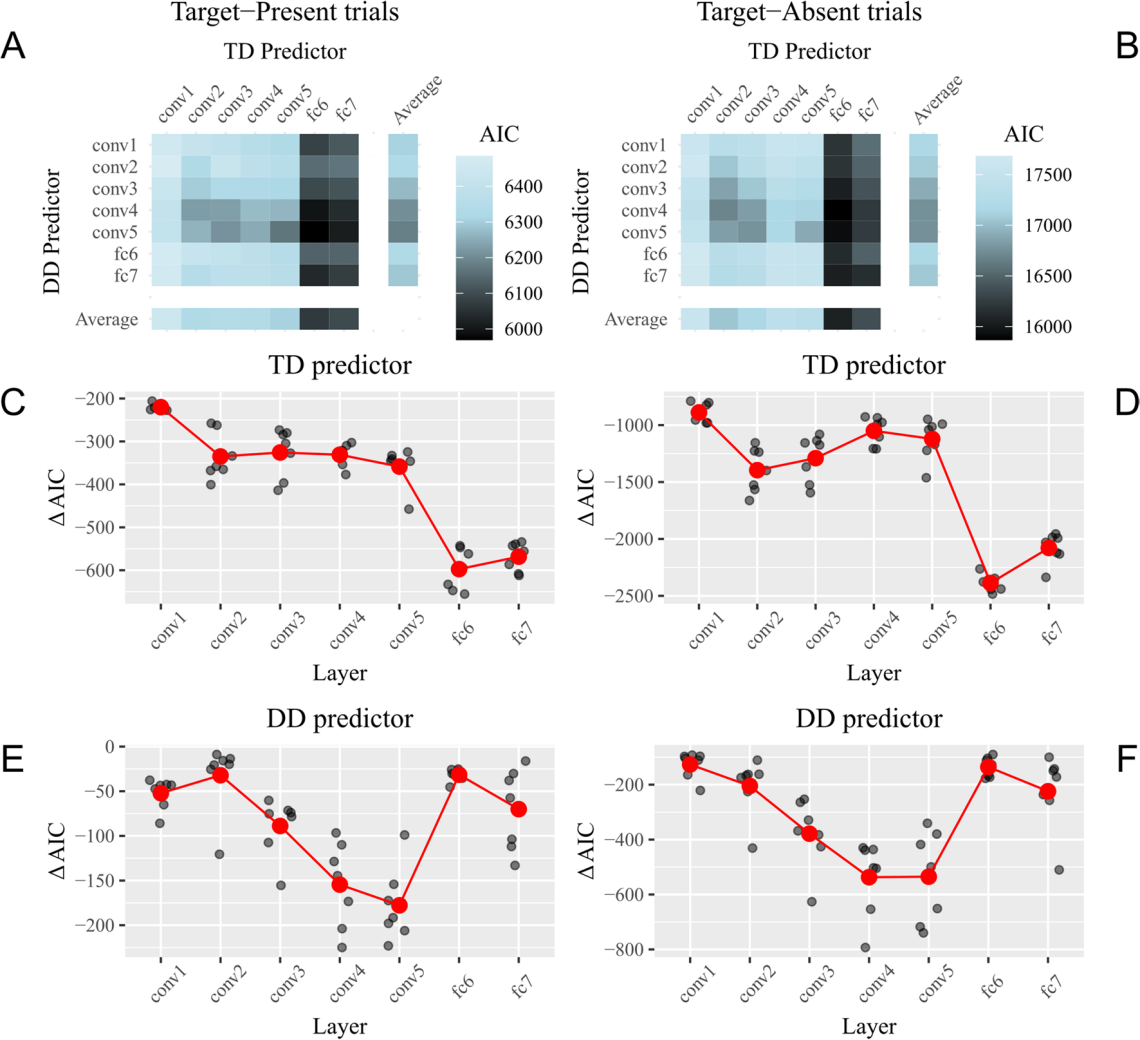
AIC and ΔAIC for the models using TD and DD similarity estimates computed from the various DCNN model layers. *Note.* The figure shows AIC and ΔAIC for the models using TD and DD similarity estimates computed from the various DCNN model layers (left panels for TP trials, right panels for TA trials). **A–B** The top panels display the total AIC for 49 models, each combining an estimate of TD similarity and one of DD similarity computed from all model layers. The colour gradient, from black to light blue, represents the AIC value of each model, with darker values (i.e., lower AIC) indicating better predictive performance. Average AIC values achieved by each TD and DD similarity predictor are also reported. **C–D** The middle panels show the change in AIC (ΔAIC) produced by adding each of the seven TD similarity predictors (estimated by one of the seven model layers) to the seven possible baseline models, with each baseline model already including one of the seven estimates of DD similarity (estimated by one of the seven model layers). Grey dots represent the ΔAIC and red dots indicate the average ΔAIC produced in the model by adding each TD predictor. **E–F** The bottom panels show the change in AIC (ΔAIC) produced by adding each of the seven DD similarity predictors to the seven possible baseline models, with each baseline model already including one of the seven estimates of TD similarity. Grey dots represent the ΔAIC, and red dots indicate the average ΔAIC produced in the model by adding each DD predictor. (Colour figure online)

In subsequent analyses, we compared TD and DD predictors across all the model layers to identify those best explaining search time (see Fig. 8).

In the analyses on TD similarity, the effect of layer was significant in both TP, *F*(7,18.414) = 278.63, *p* < 0.001, and TA, *F*(7,18.414) = 335.64, *p* < 0.001, trials. Post hoc comparison (Holm–Bonferroni corrected) revealed that the best-performing layers in TP trials were the final model layers. Indeed, the ΔAIC of TD similarity for ‘fc6’ (ΔAIC: mean =  − 597; *SD* = 48) and ‘fc7’ (ΔAIC: mean =  − 568; *SD* = 33) layers was significantly more negative than the ones from the others (all *p* values < 0.001; see Fig. 8C). This pattern was replicated in TA trials with the ΔAIC of TD similarity for ‘fc6’ (ΔAIC: mean =  − 2,387; *SD* = 74) and ‘fc7’ (ΔAIC: mean =  − 2,079; *SD* = 132) layers being more negative than ΔAIC achieved by the other layers (all *p* values < 0.001; Fig. 8D).

Also, in the analyses on DD similarity, the effect of layer was significant in both TP trials, *F*(7,18.414) = 37.091, *p* < 0.001, and TA trials, *F*(7,18.414) = 38.615, *p* < 0.001. However, post hoc comparisons (Holm–Bonferroni corrected) revealed a different pattern. In TP trials, the best-performing layers in modelling search performance were the intermediate layers ‘conv5’ (ΔAIC: mean =  − 177; *SD* = 41) followed by ‘conv4’ (ΔAIC: mean =  − 155; *SD* = 48) for which ΔAICs significantly outperformed those of the other layers (all *p* values < 0.002; see Fig. 8E). A similar pattern was found in TA trials. DD similarity computed for ‘conv4’ (ΔAIC: mean =  − 537; *SD* = 166) and ‘conv5’ (ΔAIC: mean =  − 535; *SD* = 166) significantly outperformed DD similarity for the other layers (all *p* values < 0.023; Fig. 8F).

Overall, the best-performing models are those using final layers for TD similarity and intermediate layers for DD similarity, with a normalized probability for the best-performing ones (i.e., in TP trials using TD-fc6 and DD-conv5; in TA trials using TP-fc6, DD-conv4) of *p* ≈ 0.999 to be preferred over their competitors (Wagenmakers & Farrell, 2004).

It is important to note that due to the way the experimental material was defined, the similarity distribution was optimised for the fc6 layer. Therefore, it cannot be ruled out that the procedure used may have led to underestimating similarity effects when derived from layers other than fc6. Nevertheless, the latter results indicate that the procedure did not prevent significant effects from other layers from emerging prominently and even outperforming those observed at fc6.

**Fig. 9:**
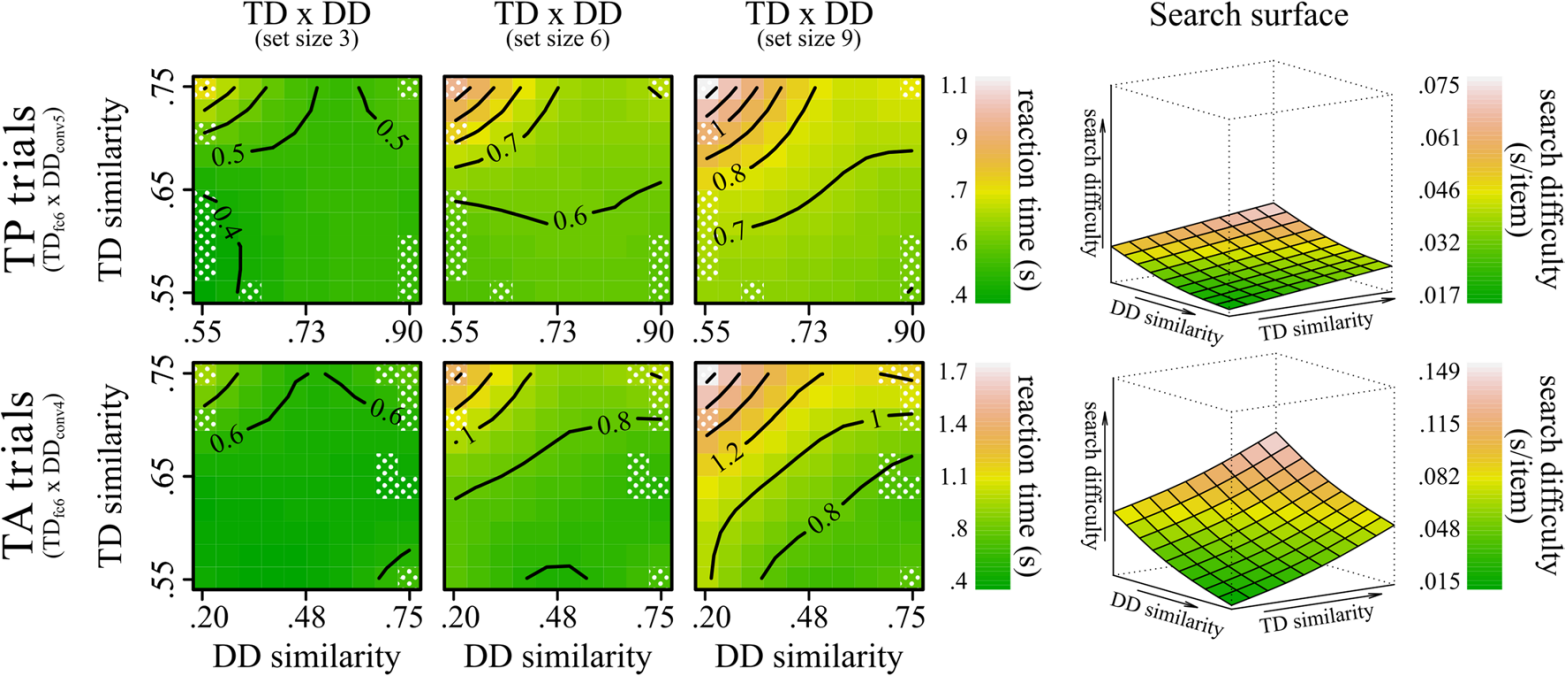
Effect of TD similarity and DD similarity on RTs as estimated by the best-performing GAMs full models. *Note.* The figure shows the effect of TD similarity and DD similarity on RTs as estimated by the best-performing GAMs full models (lines 1–2 of Table 2). The left contour plots show the effect of TD similarity (calculated via DCNN’s fc6 layer) and DD similarity (calculated via DCNN’s conv5 and conv4 layers in TP and TA trials, respectively) on RTs in TP (top graphs) and TA trials (bottom graphs) separately for the three set sizes (i.e., 3, 6, 9). Note that compared with Fig. 7, here, similarity values are not uniformly distributed since the distribution of similarity values was optimised for the fc6 layer (see Computational Framework section). Dotted areas correspond to smooth terms that are far away from data defined by the predictor variables (see documentation for vis.gam function from the mgcv package). The right 3D plots show the reconstructions of the search surface, indexing changes in search difficulty (i.e., the slope of the fitted RT × Set Size function). Plots were generated using the vis.gam function from the mgcv package. (Colour figure online)

In Table 2, we report the results for the best-performing models (using again ‘conv6’ for estimating TD similarity, but ‘conv5’ and ‘conv4’ for DD similarity in TP and TA trials, respectively). Overall, the pattern of results is similar to previous analyses. However, the interaction between DD × Set Size and DD × TD is much more pronounced and consistently significant, suggesting that previous smaller effects were due to non-optimal model layers used to estimate DD similarity. Figure 9 highlights well such interactions. At Set Size 3, the fitted RTs appear relatively flat with no clear directionality of the similarity effects except for a slight peak in RTs at the highest TD and lowest DD similarity levels. At higher set sizes, the direction of similarity effects becomes more evident. RTs slowed with increased TD and decreased DD similarity. Across set sizes, the interactive increase in TD and decrease in DD similarity leads to a noticeably nonlinear, steeper increase in RTs.

**Table 2:** Results for the primary terms of interest from each best-performing GAM model tested

Model			TD: Set Size		DD: Set Size		TD: DD		Set Size		TD		DD		
		R^2^	F	ΔR^2^	F	ΔR^2^	F	ΔR^2^	F	ΔR^2^	F	ΔR^2^	F	ΔR^2^	ƩΔR^2^
TP	_**Full**_	.387	41^***^	.003	36.3^***^	.002	37.6^***^	.005	939.3^***^	.011	198.6^***^	.014	10.2^***^	< .001	.035
TA	_**Full**_	.536	115.5^***^	.009	110.9^***^	.005	33.9^***^	.003	1486.8^***^	.025	1214.6^***^	.062	118.6^***^	.006	.110
TP	_**Set Size 3**_	.352	–	–	–	–	5.3^*^	< .001	–	–	21.5^***^	.005	1.1^n.s^	< .001	.006
TP	_**Set Size 6**_	.351	–	–	–	–	15.3^***^	.007	–	–	84.1^***^	.018	2.7^n.s^	< .001	.025
TP	_**Set Size 9**_	.348	–	–	–	–	20.8^***^	.009	–	–	98.2^***^	.022	11.9^***^	.002	.033
TA	_**Set Size 3**_	.419	–	–	–	–	10.7^***^	.004	–	–	294.9^***^	.056	27.4^***^	.005	.065
TA	_**Set Size 6**_	.48	–	–	–	–	11.9^***^	.004	–	–	494.6^***^	.086	45.9^***^	.008	.097
TA	_**Set Size 9**_	.508	–	–	–	–	13.4^***^	.004	–	–	552.4^**^*	.090	53.1^***^	.008	.103

The table reports the results for the primary terms of interest from each best-performing GAM model tested (i.e., with TD similarity consistently calculated via DCNN’s fc6 layer and DD similarity calculated via DCNN’s conv5 and conv4 layers in TP and TA trials, respectively). Rows 1 and 2 report results for the full models run separately for TP and TA trials. Rows 3 to 8 present results for the models run at each of the three set-size levels separately for TP and TA trials. For each model, the R^2^ value is reported to represent the model’s overall explained variance. Additionally, for each model term, the table provides the *F*-value and the change in unique variance (ΔR^2^), which indicates the improvement in explained variance provided by the inclusion of each specific term to the model. Finally, the column ƩΔR^2^ reports the sum of the unique variance of all terms of interest

*p < .05; **p < .01; ***p < .001; ^n.s.^ = nonsignificant

## Discussion

This study provides a method to predict human search efficiency from computational estimates of similarity between objects populating a visual scene. Using naturalistic images as stimuli, our results confirm from a more ecological perspective the fundamental similarity principles outlined by Duncan and Humphreys (1989): Search performance continuously varies across tasks and conditions and improves with decreasing TD similarity and increasing DD similarity, with these effects interacting to influence overall search efficiency. Moreover, our results confirm overall higher RTs in TA compared with TP trials, a common observation in visual search attributed to the exhaustive nature of TA trials—where all items are typically examined—and the self-terminating nature of TP trials—where the search ends as soon as the target is found, leading to greater variability in search time, which, on average, is faster (for a review, see Wolfe, 2020).

Besides supporting the search surface shape, the DCNN representations used in this study offer insight into what level of perceptual representation matters. In this regard, this study shows the effects of DD and TD similarity to be dissociated. This indicates that visual search operates at distinct levels of perceptual representation, a view that is suggested in various models and here empirically demonstrated. For example, Wolfe described two distinct levels of representational complexity at two key visual search stages. The first, ‘guidance’, extracts a coarse gist from the scene to guide attention towards the most promising items, while the second, ‘verification’, involves a more detailed visual analysis aimed at classifying each attended item as target vis-à-vis distractor (Alexander & Zelinsky, 2012; Wolfe, 1994, 2020). Likewise, recent literature further suggests that various levels of perceptual complexity are involved in visual search, ascribing them to the differential contributions of peripheral (engaged in analysing coarser information) versus foveal (responsible for detailed analysis) vision (Lleras et al., 2022; Rosenholtz, 2011).

In our study, we found that the faciliatory effects of DD similarity are best explained by the similarity between representations encoded in the intermediate DCNN layers. Conversely, we observed that the hindering effects of TD similarity are best explained by the similarity in representations encoded in the final layers of DCNN. Although it is difficult to say what type of features are encoded at the DCNN layers on which DD similarity relies, it can be said that these features are still coarse and not sufficiently complex to allow in-depth object processing for identification. Instead, the final DCNN layers on which TD similarity relies are known to encode complex features the DCNN uses to classify the objects in the images.

Drawing a parallel with the frameworks above, our findings substantiate that the similarity effects of TD vis-à-vis DD preferentially target different components of visual search. Given that the DD similarity effects are best explained by similarity across coarse perceptual representations, our findings would suggest that such similarity preferentially acts at the peripheral processing level or, within Wolfe’s framework, that such similarity might lay the most appropriate groundwork to support guidance. Conversely, considering that the most relevant similarity in the context of TD pertains primarily to the similarity between complex representations well suited for recognition, its impact is likely to be predominantly exerted during the foveal visual analysis of individual items or, within Wolfe’s framework, during verification (see also Alexander & Zelinsky, 2012). Note that this view also reconciles with early intuitions by Duncan and Humphreys (1989, 1992), who linked DD similarity to effects of local clustering of distractors through Gestalt principles of similarity based on basic features (e.g., colour or orientation). In contrast, TD effects were linked to a direct match of the input with the target template.

Importantly, these findings do not preclude the possibility that both forms of similarity still exert varying degrees of influence at multiple levels of processing. In this regard, many models of visual search posit that both types of similarity play a role early in the search process and contribute to guiding it in subsequent attentional stages (e.g., SERR model: Humphreys & Müller, 1993; Guided Search model: Wolfe, 1994). Here, we measured RTs in an overt paradigm with unlimited time to respond to match the original experiments by Duncan and Humphreys (1989). Although overall RTs provide a useful way to evaluate search efficiency, other measures derived from eye movement (Alexander & Zelinsky, 2012) or EEG (Mazza et al., 2007) are more appropriate to decompose search time into distinct components—such as guidance versus verification (Wolfe, 2020) or foveal versus peripheral processing (Lleras et al., 2022).

Future studies might consider some adaptations to our experimental design to address other relevant issues in the literature. For example, an experimental design including more set sizes might allow testing the fit for different functions known to relate RT and set size, (i.e., linear functions linked to the parallel comparison of elements to the target template or logarithmic functions denoting the serial inspection of items; Buetti et al., 2019; Z. J. Xu et al., 2021). One could also consider a different configuration in the search array, such as presenting items spread around the fixation point. This change could reduce the likelihood of clustering together near items at lower set sizes, a scenario that might prompt eye movement capture with the consequent increase in RT (Carrasco et al., 1998; Ng et al., 2018; Zelinsky & Sheinberg, 1997; but consider also crowding variations across set sizes, Lleras et al., 2022).

As mentioned, this study cannot precisely determine which features are encoded at the layers on which DD and TD similarity were found to rely. It is worth noting that DCNN biases may influence the effects we observed. Indeed, DCNNs are highly sensitive to the texture and colour of objects. Therefore, it is plausible that differences in these dimensions are effectively captured in our simulations. Less can be said about other dimensions. Future research combining our approach with feature-visualisation techniques (Zeiler et al., 2014) could better elucidate the specific features underlying similarity effects.

Finally, it is important to acknowledge that the variance explained by our variables of interest is relatively low (never exceeding ~ 11%), especially in TP trials. Lower values in TP (vis-à-vis TA) trials can plausibly be attributed to their self-terminating nature, which causes high variability in participants’ responses, thus introducing noise in trial-level analyses. Moreover, it is worth noting that a considerable degree of noise must be expected as a consequence of our ecological approach. Our experiment deliberately introduced variability with image stimuli randomly changing trial by trial. Like classical studies, here we precisely manipulated our critical variables (similarity and set size) in a controlled manner, but unlike classical studies, all other perceptual variables were left to vary naturally and randomly instead of being kept precisely constant. This approach more closely mirrors real-world conditions while inevitably influencing responses in a nonsystematic manner, thus introducing noise. Remarkably, these considerations actually support the reliability of our findings: significant similarity effects were observed *despite* this natural noise, suggesting that these are robust enough to emerge even outside a highly controlled lab-based scenario.

In conclusion, our study adopted an objective, data-driven, computational approach to independently quantify on a continuous scale the similarity between naturalistic images used as target and distractor stimuli in visual search, thus increasing ecological validity without sacrificing the control of laboratory studies. The results of this study demonstrated the validity of the approach, replicating well-established similarity effects on search performance. This indicates that this approach provides a promising ground for future research, integrating similarity analyses through vector-based deep learning techniques in visual search, akin to the successful trends observed in other research areas of cognitive psychology (Doerig et al., 2023; Günther et al., 2019; Roads & Love, 2023). Indeed, such an integration delivers a new methodological and theoretical advancement that has long been advocated in visual search (Wolfe, 2020; Wolfe & Horowitz, 2017). Finally, besides supporting the validity of the approach, our study allowed us to infer the extent to which low-level and high-level image representations are activated during the task, thereby laying the groundwork for a new approach to gain insights into the level of perceptual processing involved in visual search.

## Data Availability

Data associated with this article (i.e., the stimuli and the deidentified data) are openly available on the Open Science Framework (https://osf.io/2vad8/).

## Footnotes

1 This output layer ‘fc8’ is designed to produce the final classification and is not typically used for feature extraction due to its specificity to the 1,000 classes of ImageNet on which it is trained and its low generalizability to other tasks or datasets.

2 This concept becomes clearer when imagined in a two-dimensional space: three points equidistant from each other and from a central point can be represented by four points, one situated at the centre and the other on the perimeter of a circle, positioned at evenly spaced distances. As the ray of the circle decreases (i.e., increases TD similarity), the perimeter of the circumference decreases as well. Thus, the probability that exists three points intersecting it decreases. At the same time, as the distance between the three points decreases (i.e., increases DD similarity) again, the circumference has to decrease to keep them equidistant. Thus, the probability that exists three points that intersect the circumference decreases.

3 Note that the pattern of results remains unchanged when influential outliers are not removed (see the Supplementary Materials for details).

4 Note that the pattern of results remains unchanged when control variables are excluded from the analyses (see the Supplementary Materials for details).

## Code availability

The analysis script associated with this article is openly available on the Open Science Framework (https://osf.io/2vad8/).
